# Distinct Relationship Between Cognitive Flexibility and White Matter Integrity in Individuals at Risk of Parkinson’s Disease

**DOI:** 10.3389/fnagi.2020.00250

**Published:** 2020-08-13

**Authors:** Haidar Alzaid, Thomas Ethofer, Markus A. Hobert, Bernd Kardatzki, Michael Erb, Walter Maetzler, Daniela Berg

**Affiliations:** ^1^Department of Biomedical Magnetic Resonance, University of Tübingen, Tübingen, Germany; ^2^Department of Psychiatry and Psychotherapy, University of Tübingen, Tübingen, Germany; ^3^Department of Neurology, Christian-Albrecht University of Kiel, Kiel, Germany

**Keywords:** Parkinson’s disease, prodromal phase, diffusion tensor imaging, white matter, cognitive flexibility

## Abstract

**Background and Objective:**

Executive dysfunction is the most common cognitive impairment in Parkinson’s disease (PD), occurring even in its early stages. In our study, we applied diffusion tensor imaging (DTI) to investigate white matter integrity and its association with a specific executive function such as cognitive flexibility in individuals with risk factors for PD.

**Methods:**

We examined 50 individuals with risk factors for developing PD and 24 healthy controls from the TREND (Tübinger Evaluation of Risk Factors for Early Detection of Neurodegeneration) study including neuropsychological evaluation and DTI. Cognitive flexibility was assessed using the trail making test (TMT). Tract based spatial statistics (TBSS) were employed to assess white matter abnormalities and their correlation with cognitive flexibility.

**Results:**

TMT performance correlated with mean and axial diffusivity in several white matter regions, predominantly in the frontoparietal white matter. These effects were stronger in PD risk persons (PD-RP) than in controls as evidenced by a significant group interaction. White matter integrity and TMT performance did not significantly differ across groups.

**Conclusion:**

Based on our results, PD-RP do no exhibit white matter changes or impaired cognitive flexibility. However, specific executive functions in PD-RP are more related to white matter alterations than in healthy older adults.

## Introduction

Parkinson’s disease (PD) is a slowly progressive neurodegenerative disease characterized by an abnormal accumulation of misfolded α-synuclein protein in the brain. Clinically, it is diagnosed by the cardinal motor symptoms bradykinesia, resting tremor, and rigidity (Tolosa et al., 2006). It is well documented, that the motor symptoms of PD are caused by cell death in the substantia nigra. However, studies have shown that the onset of motor manifestations of PD, which is required for the clinical diagnosis, starts only after at least 50% of the neurons in substantia nigra have died (Fearnley and Lees, 1991), and thus it has been suggested that neurodegeneration starts several years before the development of the cardinal motor symptoms (Miller and O’Callaghan, 2015). This time period preceding the clinical diagnosis is called the prodromal phase. During this phase, individuals at risk of developing PD can exhibit non-motor symptoms including hyposmia, rapid eye movement (REM)-sleep behavior disorder (RBD), depression, autonomic dysfunction, and mild cognitive impairment (Berg et al., 2015).

Mild cognitive impairment, in particular executive dysfunction, is commonly reported throughout all stages of PD (Litvan et al., 2011). Furthermore, a recent study revealed that executive function is the most affected cognitive domain in the prodromal phase of PD, and might be a useful non-motor biomarker for this phase (Fengler et al., 2017). There is cumulative evidence for the involvement of the frontoparietal network in executive functions (Collette et al., 2006) and it has been suggested that cortical “disconnection” through a disturbance of intra- and interhemispheric white matter connections, especially in the anterior white matter, represents a possible mechanism underlying age-related executive dysfunction (O’Sullivan et al., 2001) as well as disease-related alterations in PD (Bledsoe et al., 2018).

Although the death of dopaminergic neurons in the substantia nigra is considered a main pathomechanism of PD (Lees et al., 2009), several neuropathological studies revealed that axonal dysfunction and degeneration in diverse brain (Cheng et al., 2010), and even peripheral (Knudsen et al., 2018) regions are an additional important hallmark in the pathophysiology of PD. In fact, axonal degeneration may be the earliest sign of the disease, occurring long before the death of neuron cell bodies (Cheng et al., 2010). Consequently, diffusion tensor imaging (DTI; Basser et al., 1994) was successfully applied to detect alterations of structural fiber connectivity in PD (Atkinson-Clement et al., 2017; Koirala et al., 2018, 2019) including early stages (Zhang et al., 2015) and also identified candidate white matter structures for cognitive impairment in PD (Melzer et al., 2013; Zheng et al., 2014). Fractional anisotropy (FA) and mean diffusivity (MD) are the DTI-derived parameters that are primarily used to detect white matter microstructural alterations. FA is a measure of orientational coherence along the fibers, while MD is a measure of averaged diffusivity, representing the amount of diffusion in all directions (Pierpaoli and Basser, 1996). Typically, white matter pathology is associated with decreased FA and increased MD. In addition, axial diffusivity (AD) and radial diffusivity (RD) have been proven useful in detecting axonal damage and myelin loss, respectively (Song et al., 2002; Sun et al., 2006). AD reflects diffusion along the main axis (λ_1_), whereas RD reflects perpendicular diffusion to the main axis (λ_2_, λ_3_).

In the current study, we investigated whether alterations of fiber connectivity as revealed by DTI are already detectable in individuals with risk factors for developing PD. Specifically, we utilized tract-based spatial statistics (TBSS) to investigate the following hypotheses:

1. White matter pathology is an early sign of PD and can already be detected in the prodromal phase.
2. Impairment of specific executive functions as assessed by the trail making test (TMT) are related to white matter pathology in individuals with risk factors for PD.

## Materials and Methods

### Participants

This cross-sectional study investigated participants from the TREND study (Tübinger Evaluation of Risk Factors for Early Detection of Neurodegeneration), a prospective biannual follow-up study that aims at identifying risk factors for the development of PD. Sociodemographic and clinical data are presented in Table 1. Fifty individuals with well-known risk factors for developing PD (PD risk persons, PD-RP, 19 males, 31 females, and average age 66.1 ± 6.4 years), and 24 controls (controls, 14 males, 10 females, and average age 68.3 ± 6.5 years) from the TREND cohort were included in the current study. Study details and recruitment criteria have been previously published (Gaenslen et al., 2014). All participants were older than 50 years, were able to walk without aid, had no diagnosis of any neurodegenerative disease, no history of stroke, inflammatory diseases of the central nervous system, or polyneuropathy, were free of antipsychotic or other antidopaminergic drugs, and exhibited no significant impairment of vision or hearing. PD-RP had at least one of the following diagnoses:

**TABLE 1:** Demographics and clinical parameters of the cohorts.

	Control (*n* = 24)	PD-RP (*n* = 50)	*P*-Value
Gender (m/f)	14/10	19/31	0.1^*a*^
Age (y)*	68 ± 6	66 ± 6	0.19^*b*^
Years of education (range)	15 (10–20)	15 (10–21)	0.89^*b*^
Depression (prevalence)	0%	82%	–
Hyposmia (prevalence)	0%	28%	–
RBD (prevalence)	0%	32%	–
UPDRS-III (range)	1 (0–5)	1 (0–9)	0.75^*c*^
Sniffin‘ sticks (range)	13 (10–15)	10 (3–16)	0.003^c^**
RBDSQ (range)	2 (0–6)	4 (0–10)	0.006^c^**
BDI-II (range)	3 (0–9)	10 (0–42)	0.00009^c^**
MMSE (range)	29 (24–30)	29 (25–30)	0.55^*c*^
TMT A (s)*	34 ± 9	35 ± 11	0.65^*c*^
TMT B (s)*	76 ± 25	76 ± 28	0.94^*c*^
TMT B-A (s)*	42 ± 21	41 ± 24	0.78^*b*^

*Values are expressed as the mean ± standard deviation. **corrected for multiple comparisons. Abbreviations: BDI-II, Beck Depression Inventory II; MMSE, Mini Mental State Examination; TMT, Trail making test; and UPDRS-III, Motor part of the Unified Parkinson’s Disease Rating Scale. ^*a*^*X*^2^-test. ^*b*^Independent sample *t*-test. ^*c*^Mann–Whitney-*U* test.

– Hyposmia, assessed using Sniffin’ sticks (Burghardt Medizintechnik, Wedel, Germany) with a set consisting of 16 different odors, identifying < 10 of odors is indicative of impaired sense of smell (Hummel et al., 2007).
– REM sleep RBD, assessed using the RBD Screening questionnaire (RBDSQ), a value of 5 out of 10 possible items was considered was a positive test result, which can indicate the presence of RBD with a sensitivity of 96% and a specificity of 56% (Stiasny-Kolster et al., 2007).
– Depression, defined as current depression or the occurrence of at least one major depressive episode during their lifetime according to ICD-10 and DSM-IV criteria.

Individuals in the control group received the same screening and were explicitly chosen to not have any risk factors for PD.

Additionally, the motor part of the Unified Parkinson’s Disease Rating Scale (UPDRS-III) and Beck Depression Inventory II (BDI-II; Beck et al., 1996) were administered to evaluate motor and depressive symptoms, respectively, though not considered in the selection of PD-RP.

### Neuropsychology

All participants underwent testing of cognitive flexibility based on the TMT (Reitan and Skills, 1958) consisting of part A and B. In part A, subjects have to connect numbers from 1 to 25. It is used to test visual search ability and motor speed. In part B, subjects have to connect numbers and letters in an alternating way (1-A-2-B-3-C….). In addition to the components from part A, part B also tests cognitive flexibility and working memory. We chose the derived score B-A as our parameter of interest, as (Sanchez-Cubillo et al., 2009) have recommended using B-A to minimize the influence of motor speed, visuospatial perception, and working memory demands to provide a more reliable indicator of cognitive flexibility. Additionally, the Mini-Mental State Examination (MMSE) was carried out in all participants (Folstein et al., 1975).

### Image Acquisition

Structural T1-weighted images (TR = 2300 ms, TE = 4.18 ms, TI = 900 ms, and voxel size: 1 mm × 1 mm × 1 mm) were acquired with a 3 T scanner (Siemens PRISMA, Erlangen, Germany). Diffusion-weighted images were acquired using a “Stejskal-Tanner” sequence (TR = 6.0 s, TE = 69 ms, flip angle = 90°, 50 axial slices, and 2 acquisitions) with a voxel size of 1.7 × 1.7 × 2.5 mm^3^ along 30 independent directions using a *b*-value of 1000 s/mm^2^. Additionally, 12 images with a *b*-value of 0 s/mm^2^ were acquired throughout the sequence.

### DTI Data Analysis

Data preprocessing and DTI analysis were carried out using FSL 5.0.9 (FMRIB Software Library, http://fsl.fmrib.ox.ac.uk/fsl/). Preprocessing included correction of eddy current distortions and motion artifacts (Andersson and Sotiropoulos, 2016). Head motion was compared between groups across 6 direction (translation *x*/*y*/*z* and rotation *x*/*y*/*z*). The DTIFIT tool was used to fit a diffusion tensor model at each voxel and generate FA, MD maps, and the eigenvalues (λ_1_, λ_2_, λ_3_). Axial (AD = λ_1_) and radial [RD = (λ_2_ + λ_3_)/2] diffusivity maps were calculated from these eigenvalues. Voxel-wise statistical analysis of the FA data was carried out using TBSS included in FSL (Smith et al., 2004, 2006). FA data from each subject was aligned into a common space using the non-linear registration tool FNIRT (Andersson et al., 2007), which uses a *b*-spline representation of the registration warp field (Rueckert et al., 1999), and finally averaged to create a mean FA image. The mean FA image was then thinned to create a mean FA skeleton which represents the centers of all tracts common to the group; an FA threshold of 0.2 was used for the skeleton. Each subject’s aligned FA data was then projected onto this skeleton. The same FA transformation was applied to MD, RD, and AD maps, and projected onto the same skeleton. Statistical models were set up to enable comparison across groups as well as correlation with TMT B-A and included age and gender as covariates of no interest. Additionally, we ran correlation analyses with the risk measures BDI-II, RBDSQ, and Sniffin’ sticks across both groups and within each group to determine their influence on white matter integrity. Using the “Randomize” tool incorporated in FSL we ran non-parametric permutation-based statistical tests on the DTI maps with 10000 permutations and threshold-free cluster enhancement (Smith and Nichols, 2009) to correct for multiple comparisons across the whole brain at a significance threshold of *P* < 0.05 using family-wise error correction.

### Analysis of White Matter Integrity and Cognitive Flexibility

**FIGURE 1:**
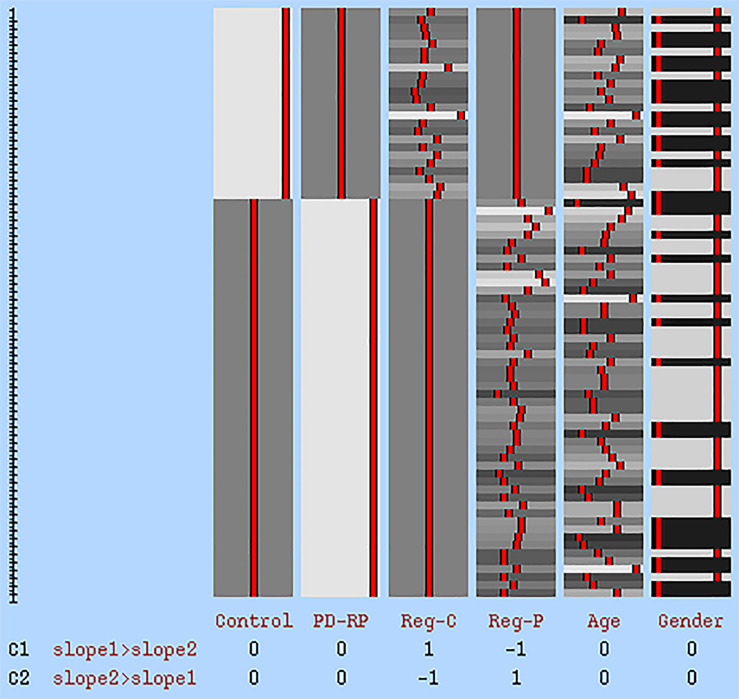
GLM statistical model for group interaction effect. The first two columns represent tbss data of each group. Reg-C and Reg-P represent the regressor for the control group and PD-RP, respectively. Age and gender were added as covariates of no interest. C1: higher effect in control, C2: higher effect in PD-RP.

The relationship between white matter integrity and cognitive flexibility was explored using the abovementioned tbss method. A correlation analysis between DTI parameters and TMT B-A was carried out to investigate overall correlation across both groups and within each group. Additionally, the group interaction effect was examined using the FSL GLM model “Two Groups with continuous covariate interaction” (see Figure 1). Age and gender were added as covariates of no interest in every correlation model. The same tbss settings as described above were applied in the correlation analyses. Clusters demonstrating a significant group interaction were defined based on Johns Hopkins University’s Mori white matter atlas (Mori et al., 2008). DTI parameters in each significant cluster were than extracted from the skeletonized tbss image of each participant and plotted against their TMT B-A score. As a safeguard we ran all our analyses by leaving out the most extreme data point. While this obviously resulted in different statistical values it had no impact on statistical inference (i.e., whether results were statistical significant or not).

### Statistical Analysis

Statistical analysis of the clinical and neuropsychological measures was carried out using SPSS version 25 software (SPSS Inc, Chicago, IL, United States). Independent sample *t*-tests as well as Mann–Whitney-*U* tests were used to compare mean/median scores between the groups for normally and non-normally distributed parameters, respectively. The *X*^2^ test was used to assess potential differences in gender distribution. Partial Correlation analysis between TMT B-A, BDI-II, RBDSQ, and Sniffin’ sticks scores controlling for age and gender was used to evaluate, whether TMT B-A performance was driven by one of the risk factors depression, RBD or hyposmia. A non-corrected *P*-value < 0.05 was considered significant. There was no significant difference in head motion between groups across all 6 directions (see Supplementary Material).

## Results

### Demographics and Clinical Features

Age, gender and education as well as UPDRS-III and MMSE scores were not significantly different between PD-RP and controls. As expected, BDI-II, RBDSQ, and Sniffin’ sticks scores were significantly higher in PD-RP than in controls. The TMT B-A score was not significantly different between the groups and did not significantly correlate with BDI-II, RBDSQ or Sniffin’ sticks scores.

### TBSS Analysis

**FIGURE 2:**
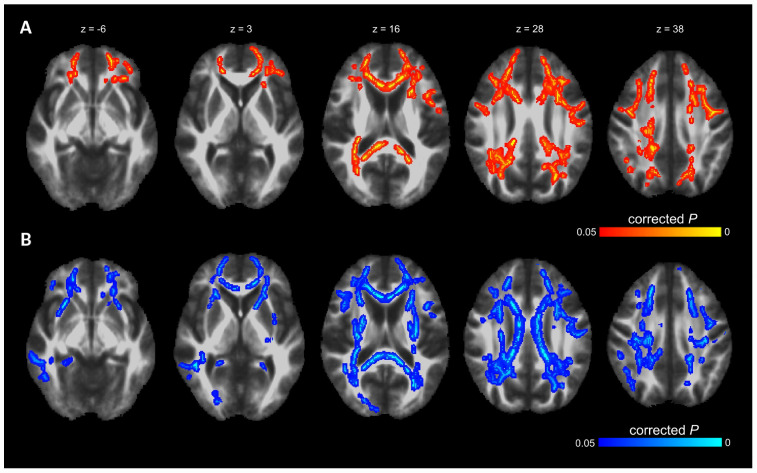
The results of TBSS whole-brain regression analysis overlaid on mean FA (1 mm) template and presented in radiological convention. **(A)** Significant group interaction (PD-RP > controls) determined in the regression analysis between cognitive flexibility (TMT B-A) and mean diffusivity (MD). **(B)** Significant group interaction (PD-RP > controls) determined in the regression analysis between cognitive flexibility (TMT B-A) and axial diffusivity (AD).

**FIGURE 3:**
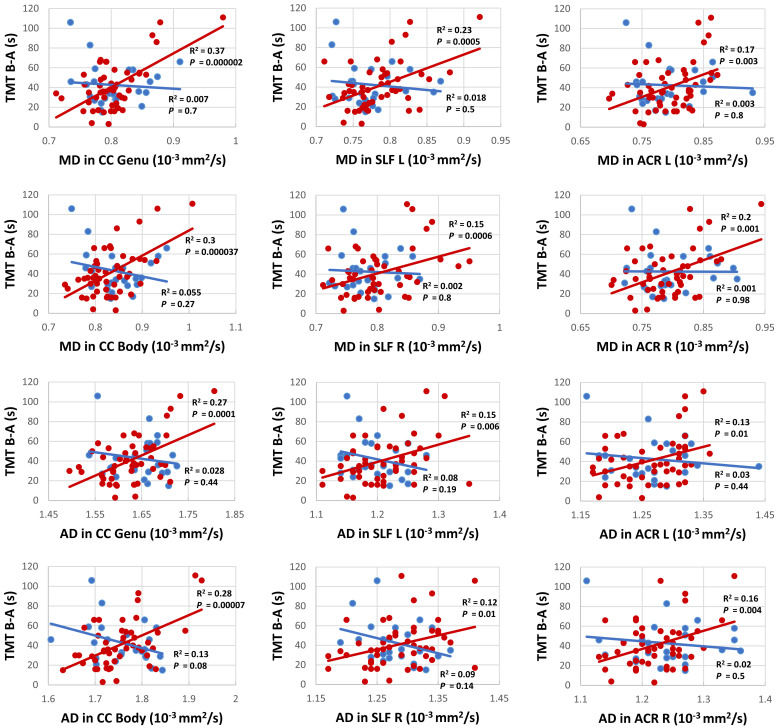
Correlation analysis between TMT B-A and MD (upper six panels) as well as TMT B-A and AD (lower six panels) for persons with (red) and without (blue) risk factors for PD for the clusters with the highest correlation in commissural tracts: genu and body of the corpus callosum (CC), projection tracts: left and right anterior corona radiata (ACL), and association tracts: left and right superior longitudinal fasciculus (SLF).

Voxel-wise TBSS whole brain analysis did not reveal any significant difference between both groups controlling for multiple comparisons across the whole brain. The correlation analysis between TMT B-A performance and diffusion parameters within the PD-RP group, however, revealed significant effects for MD and AD in several brain areas including, commissural tracts, e.g., corpus callosum, association tracts, e.g., superior longitudinal fasciculus and projection tracts, e.g., corona radiata (for full results see Figures 2, 3 and Tables 2, 3). These effects were stronger in PD-RP than in controls as evidenced by a significant group interaction (*P* < 0.05 FWE-corrected, see Figures 2, 3). The three risk measures BDI-II, RBDSQ and Sniffin’ sticks scores did not significantly correlate with diffusion parameters in each group.

**TABLE 2:** MD clusters with a significant group interaction.

Region		Cluster size (voxels)	Individuals at risk of PD		Control group	
				
			MD ± SE (10^–3^ mm^2^/s)	*R*^2^/*P*	MD ± SE (10^–3^ mm^2^/s)	R^2^/*P*
ACR	Left	722	0.795 ± 0.006	0.168/0.003	0.793 ± 0.010	0.003/0.80
	Right	410	0.791 ± 0.007	0.200/0.001	0.797 ± 0.011	0.001/0.98
SCR	Left	153	0.786 ± 0.005	0.067/0.07	0.788 ± 0.010	0.027/0.44
	Right	220	0.778 ± 0.005	0.136/0.008	0.769 ± 0.008	0.014/0.59
PCR	Left	57	0.827 ± 0.006	0.130/0.01	0.810 ± 0.008	0.009/0.66
	Right	231	0.856 ± 0.007	0.166/0.003	0.847 ± 0.010	0.002/0.85
CC	Genu	435	0.802 ± 0.006	0.373/0.000002	0.804 ± 0.009	0.007/0.7
	Body	516	0.831 ± 0.007	0.301/0.000037	0.845 ± 0.011	0.055/0.27
	Splenium	884	0.758 ± 0.004	0.230/0.0004	0.755 ± 0.006	0.157/0.06
SLF	Left	206	0.784 ± 0.006	0.225/0.0005	0.773 ± 0.008	0.018/0.54
	Right	116	0.801 ± 0.008	0.149/0.0006	0.785 ± 0.008	0.002/0.82
ALIC	Left	22	0.757 ± 0.008	0.061/0.083	0.755 ± 0.013	0.009/0.66
RIC	Right	53	0.764 ± 0.006	0.156/0.0045	0.752 ± 0.008	0.044/0.32
EC	Left	33	0.801 ± 0.007	0.057/0.094	0.786 ± 0.012	0.015/0.56
SFOF	Left	7	0.795 ± 0.012	0.024/0.278	0.791 ± 0.020	0.018/0.53
PTR	Right	98	0.897 ± 0.009	0.216/0.0007	0.900 ± 0.010	0.020/0.51
T	Right	17	0.942 ± 0.013	0.163/0.0036	0.945 ± 0.019	0.003/0.79

*All Values are expressed as the mean ± standard error (SE). Abbreviations: MD, mean diffusivity; ACR, anterior corona radiata; SCR, superior corona radiata; PCR, posterior corona radiata; CC, corpus callosum; SLF, superior longitudinal fasciculus; ALIC, anterior limb of internal capsule; RIC, retrolenticular part of internal capsule; EC, external capsule; SFOF, superior fronto-occipital fasciculus; PTR, posterior thalamic radiation; and T, tapetum.*

**TABLE 3:** AD clusters with a significant group interaction.

Region		Cluster size (voxels)	Individuals at risk of PD		Control group	
				
			AD ± SE (10^–3^ mm^2^/s)	*R*^2^/*P*	AD ± SE (10^–3^ mm^2^/s)	*R*^2^/*P*
ACR	Left	841	1.264 ± 0.007	0.126/0.011	1.268 ± 0.012	0.028/0.44
	Right	860	1.221 ± 0.007	0.161/0.0038	1.244 ± 0.014	0.024/0.47
SCR	Left	492	1.253 ± 0.009	0.052/0.112	1.250 ± 0.014	0.028/0.43
	Right	915	1.196 ± 0.008	0.045/0.137	1.191 ± 0.012	0.071/0.21
PCR	Left	271	1.349 ± 0.009	0.214/0.0007	1.338 ± 0.012	0.039/0.36
	Right	401	1.350 ± 0.010	0.152/0.005	1.342 ± 0.014	0.015/0.57
CC	Genu	812	1.627 ± 0.009	0.270/0.0001	1.647 ± 0.011	0.028/0.44
	Body	1404	1.755 ± 0.009	0.283/0.00007	1.759 ± 0.013	0.131/0.08
	Splenium	1647	1.684 ± 0.009	0.162/0.0038	1.684 ± 0.012	0.156/0.06
SLF	Left	304	1.210 ± 0.007	0.148/0.006	1.195 ± 0.008	0.078/0.19
	Right	468	1.285 ± 0.008	0.119/0.014	1.282 ± 0.009	0.095/0.14
ALIC	Left	177	1.372 ± 0.011	0.151/0.005	1.377 ± 0.014	0.016/0.56
	Right	91	1.306 ± 0.009	0.070/0.06	1.321 ± 0.012	0.025/0.46
PLIC	Left	76	1.411 ± 0.009	0.020/0.327	1.403 ± 0.011	0.036/0.37
	Right	125	1.414 ± 0.008	0.043/0.146	1.412 ± 0.009	0.080/0.18
RIC	Left	67	1.498 ± 0.007	0.026/0.26	1.495 ± 0.012	0.085/0.17
	Right	140	1.445 ± 0.011	0.061/0.08	1.472 ± 0.019	0.013/0.6
EC	Left	404	1.251 ± 0.007	0.066/0.07	1.243 ± 0.009	0.158/0.06
	Right	233	1.319 ± 0.007	0.093/0.03	1.311 ± 0.011	0.109/0.16
SFOF	Left	9	1.288 ± 0.002	0.066/0.07	1.283 ± 0.002	0.002/0.82
	Right	31	1.225 ± 0.010	0.063/0.08	1.236 ± 0.010	0.119/0.1
PTR	Left	88	1.672 ± 0.012	0.045/0.14	1.675 ± 0.014	0.231/0.02
	Right	205	1.622 ± 0.010	0.071/0.06	1.636 ± 0.015	0.184/0.04
T	Left	2	1.596 ± 0.002	0.101/0.024	1.590 ± 0.003	0.001/0.92
	Right	18	1.672 ± 0.015	0.121/0.013	1.665 ± 0.019	0.017/0.55
SS	Right	15	1.571 ± 0.012	0.006/0.6	1.607 ± 0.020	0.096/0.14
UF	Right	3	1.515 ± 0.014	0.054/0.1	1.512 ± 0.017	0.037/0.37
CG	Right	45	1.400 ± 0.009	0.063/0.08	1.410 ± 0.012	0.047/0.31
CH	Right	42	1.325 ± 0.013	0.075/0.055	1.317 ± 0.021	0.057/0.26

*All Values are expressed as the mean ± standard error (SE). Abbreviations: AD, axial diffusivity; ACR, anterior corona radiata; SCR, superior corona radiata; PCR, posterior corona radiata; CC, corpus callosum; SLF, superior longitudinal fasciculus; ALIC, anterior limb of internal capsule; RIC, retrolenticular part of internal capsule; EC, external capsule; SFOF, superior fronto-occipital fasciculus; PTR, posterior thalamic radiation; T, tapetum; PLIC, posterior limb of internal capsule; CH, cingulum of hippocampus; CG, cingulate gyrus; UF, uncinate fasciculus; and SS, sagittal stratum.*

## Discussion

In recent years, enormous efforts have been devoted to better understand the time before motor symptoms allow clinical diagnosis of PD, i.e., the prodromal phase (Berg et al., 2015). Nevertheless, the number of neuroimaging studies on white matter alterations in this phase is still very limited with most studies focusing on idiopathic RBD as a predictor of α-synucleinopathies (Unger et al., 2010; Scherfler et al., 2011; Rahayel et al., 2015), and not the broad definition of prodromal PD (Berg et al., 2015). This study is, to our best knowledge, the first to reveal a correlation between white matter architecture and a cognitive function, such as cognitive flexibility, in PD-RP.

We found no significant differences between the two groups regarding the diffusion parameters arguing against a general application of the examined diffusion parameters as early marker for the development of PD. In contrast, a study on a PD-RP cohort with substantia nigra hyperechogenicity and/or hyposmia, revealed increased MD in the posterior thalamus, inferior longitudinal fasciculus, fornix and the corticospinal tract in the PD-RP group, but failed to demonstrate a significant difference between PD patients and controls (Heldmann et al., 2018). The authors suggested that PD-RP/PD patients with hyposmia could have a more severe disease course with a different pattern of neurodegeneration in comparison to those without hyposmia. This may explain the negative results in our study, since only 28% of our PD-RP cohort presented with hyposmia. On the other hand, a study on G2019S LRRK2 mutation carriers, an asymptomatic cohort generally considered at higher risk of PD, did not reveal any significant structural brain changes using DTI and VBM (voxel-based morphometry). However, a tendency for higher FA and lower MD in the mutation carriers was observed, which may hint at the involvement of a compensatory mechanism. Similarly, a recent study using graph-theory revealed greater local connectivity in regions relating to motor, olfactory, and sleep functions in PD-RP, suggesting neural compensation in the prodromal phase (Wen et al., 2017). This discrepancy between studies on PD-RP could be due to the application of different recruitment criteria, as it should be noted that our definition of the PD-RP group implicates that only part of this group will develop PD, so a true effect may be driven by a large proportion of non-converters. Based on this assumption it is even more interesting that our analysis revealed a significant correlation between TMT results and MD in the superior longitudinal fasciculus connecting the frontal and parietal cortex (Schmahmann et al., 2008), as well as the corpus callosum and multiple projection tracts, such as the corona radiata, while no such effects were observed for FA.

This observation is in line with MRI studies on early PD demonstrating that alterations of MD precede FA reduction (Melzer et al., 2013) as well as gray matter (Duncan et al., 2016). Also in line with our result, MD was found to be associated with executive function in PD, while no significant association was found for FA (Melzer et al., 2013). This is in contrast to studies on aging that demonstrated a positive correlation between FA and executive function (Perry et al., 2009; Halliday et al., 2019) and suggests that distinct white matter pathologies are responsible for cognitive impairments related to healthy aging and PD. A similar finding was also reported in individuals with traumatic brain injury (Kinnunen et al., 2011).

Our analyses also revealed a significant correlation between TMT performance and AD in several frontoparietal white matter regions. Although the mechanism causing increases of AD is not fully understood, it has been implicated as an early state-specific marker with a high sensitivity for early neurodegeneration and cognitive impairment (Acosta-Cabronero et al., 2012; Molinuevo et al., 2014). It has also been suggested that increased AD may represent an upstream event preceding axonal degeneration, e.g., inflammation (Acosta-Cabronero et al., 2012). In addition, the involvement of the frontoparietal network as an essential neurobiological correlate of executive functions concurs with previous DTI (Halliday et al., 2019) and functional magnetic resonance imaging (fMRI; Niendam et al., 2012) studies in healthy individuals as well as fMRI (Lewis et al., 2003) and DTI (Melzer et al., 2013) studies on neural substrates underlying impairments of executive functions in PD. Similarly, microstructural alterations in the corpus callosum and their association with mild cognitive impairment in PD including executive function impairments have been reported (Bledsoe et al., 2018), adding to the growing body of evidence suggesting both intra- and interhemispheric “disconnection” as a possible mechanism of executive dysfunction in PD (Melzer et al., 2013; Zheng et al., 2014).

It should be noted that the correlation between TMT performance and MD as well as AD was only found in the group of PD-RP, but not in the control group. An analogous finding has been reported in a DTI study on executive dysfunctions in individuals with traumatic brain injury with a significant correlation between TMT B-A performance and RD in frontoparietal areas which was restricted to the patient group, but not found in healthy individuals (Kinnunen et al., 2011). This distinct relationship may indicate that deficits in specific executive functions in PD-RP are more related to white matter pathology than in healthy older adults.

It is worth mentioning that PD-RP did not differ from the control group in regards to their TMT performance. While other imaging studies also failed to demonstrate cognitive deficits in PD-RP (Wen et al., 2017; Heldmann et al., 2018), a recent review reported the existence of executive function impairments in prodromal PD (Fengler et al., 2017). Hence, prospective longitudinal studies are still needed to determine the role of cognitive changes as a reliable biomarker for prodromal PD.

Interestingly, two fMRI-studies have reported a higher task-related activity in several cerebral regions in G2019S LRRK2 mutation carriers while maintaining an adequate executive function performance, suggesting a neural compensatory mechanism that enables intact cognitive performance (Thaler et al., 2013; Bregman et al., 2017).

Our study revealed no correlation between DTI measure and risk scores, such as sniffin’ sticks and RBDSQ. This is in contrast to previous studies on PD-RP revealing structural correlates of olfactory tests (Wen et al., 2017; Heldmann et al., 2018) as well as RBDSQ (Wen et al., 2017). This is may be due to the lower prevalence of these risk factors in our PD-RP cohort.

This study faces some limitations. For example, this is a cross-sectional study and we do not have MRIs from the entire TREND cohort (which has already observed 16 PD converters within its lifetime). Thus, our results are built on a PD-RP cohort and it is not clear how many participants will eventually convert to PD. This limitation is shared with many other studies in the field. Due to this limitation we can also not exclude that the effect observed in this study is a constitutional factor, rather than a dynamic process indicating prodromal PD (Mirelman et al., 2011). This aspect has to be further investigated in longitudinal prospective cohorts. Another limitation is the relatively low specificity of our selected prodromal markers. Individuals with a positive score on RBDSQ and hyposmia may have prodromal PD with a likelihood of 2.8 and 6.4, respectively. While our most frequent risk factor, depression, has an estimated likelihood of only 1.6 (Heinzel et al., 2019). Thus, a prodromal risk score based on multiple prodromal markers would be a more appropriate approach to categorize PD-RP and yield a higher probability for prodromal PD.

To avoid a loss of sensitivity due to multiple testing we focussed on a single measure of executive functioning, namely the cognitive flexibility, which may be a plausible explanation for absence of a group difference in TMT. Therefore, future imaging studies on PD-RP should apply a more comprehensive executive function screening in addition to other cognitive domains to get a better understanding of the structural correlates of the cognitive deficits in prodromal PD.

The severity of depressive symptoms, as measured by BDI-II, did not correlate with TMT performance or white matter integrity in our analysis. Given that most of our PD-RP group have had at least one depressive episode during their lifetime, we cannot exclude the possibility that our result is a lingering effect of depression, which can be only partially accounted for by the severity of current depressive symptoms (Rock et al., 2014).

In summary, DTI and cognitive flexibility as assessed by TMT were not able to reliably differentiate PD-RP from healthy controls and thus may lack the needed sensitivity as biomarkers for the prodromal phase of PD. Nevertheless, DTI revealed frontoparietal white matter alterations related to a specific executive function only in PD-RP, which may indicate that deficits in specific executive functions PD-RP are more related to white matter alterations than in healthy older adults.

## Data Availability Statement

The datasets generated for this study are available on request to the corresponding author.

## Ethics Statement

The studies involving human participants were reviewed and approved by the Ethical Committee of the Medical Faculty at the University of Tübingen (Nr. 90/2009BO2). The participants provided their written informed consent to participate in this study.

## Author Contributions

DB and WM contributed conception and design of the study. DB, MH, TE, and WM organized the study. HA performed data analysis. BK and ME provided analysis tools. HA wrote the first draft. DB, TE, and WM wrote sections of the manuscript. All authors contributed to manuscript revision, read and approved the submitted version.

## Conflict of Interest

The authors declare that the research was conducted in the absence of any commercial or financial relationships that could be construed as a potential conflict of interest.
