# Supplementary material for: Distinct Relationship Between Cognitive Flexibility and White Matter Integrity in Individuals at Risk of Parkinson’s Disease
Source: Front Aging Neurosci. 2020 Aug 13;12:250. doi: 10.3389/fnagi.2020.00250 (PMC7439016; doi:10.3389/fnagi.2020.00250)
Supplement: Supplementary file 1 [file Table_1.DOCX]

Comparison of head motion:

| Parameter | PD-RP | Controls |
| --- | --- | --- |
| Translation [x] (mm) | -0.038 | -0.053 |
| Translation [y] (mm) | -0.32 | -0.31 |
| Translation [z] (mm) | -0.07 | -0.31 |
| Rotation [x] (radian) | 0.1 * 10^-3^ | 0.3 * 10^-3^ |
| Rotation [y] (radian) | -2.4 * 10^-3^ | -1.9 * 10^-3^ |
| Rotation [z] (radian) | -1.1 * 10^-3^ | -2.6 * 10^-3^ |

There is no significant difference between groups across the six parameters at a non-corrected *P*-value of 0.05.
